# A Global Survey of Gene Regulation during Cold Acclimation in *Arabidopsis* *thaliana*

**DOI:** 10.1371/journal.pgen.0010026

**Published:** 2005-08-19

**Authors:** Matthew A Hannah, Arnd G Heyer, Dirk K Hincha

**Affiliations:** 1 Max-Planck-Institut für Molekulare Pflanzenphysiologie, Potsdam, Germany; 2 Biologisches Institut, Abteilung Botanik, Universität Stuttgart, Stuttgart, Germany; Stanford University School of Medicine, United States of America

## Abstract

Many temperate plant species such as *Arabidopsis thaliana* are able to increase their freezing tolerance when exposed to low, nonfreezing temperatures in a process called cold acclimation. This process is accompanied by complex changes in gene expression. Previous studies have investigated these changes but have mainly focused on individual or small groups of genes. We present a comprehensive statistical analysis of the genome-wide changes of gene expression in response to 14 d of cold acclimation in *Arabidopsis,* and provide a large-scale validation of these data by comparing datasets obtained for the Affymetrix ATH1 Genechip and MWG 50-mer oligonucleotide whole-genome microarrays. We combine these datasets with existing published and publicly available data investigating *Arabidopsis* gene expression in response to low temperature. All data are integrated into a database detailing the cold responsiveness of 22,043 genes as a function of time of exposure at low temperature. We concentrate our functional analysis on global changes marking relevant pathways or functional groups of genes. These analyses provide a statistical basis for many previously reported changes, identify so far unreported changes, and show which processes predominate during different times of cold acclimation. This approach offers the fullest characterization of global changes in gene expression in response to low temperature available to date.

## Synopsis

Freezing tolerance is an important determinant of geographical distribution of plant species, and freezing damage in crop plants leads to severe losses in agriculture. Many temperate plants increase their freezing tolerance during exposure to low, but nonfreezing temperatures, a process known as cold acclimation. Freezing tolerance and cold acclimation are complex, quantitative genetic traits. The number and functional roles of the responsible genes are not known for any plant species. Using the model plant *Arabidopsis thaliana,* which is moderately freezing tolerant and able to cold acclimate, the global regulation of gene expression during exposure to 4 °C for 14 d was analyzed by microarray hybridization. For validation of gene expression data, triplicate biological samples were hybridized to two different oligonucleotide arrays. Results from the two platforms showed good agreement, indicating the reliability of the measurements. The authors combined their data with all publicly available data on cold-regulated gene expression in *A. thaliana* to compile a database detailing the cold responsiveness of 22,043 genes as a function of exposure time. In addition, thorough statistical analysis was used to identify metabolic pathways and physiological processes that are predominantly involved in the plant cold-acclimation process.

## Introduction

Cold has major influences on crop production, limiting geographical distribution and growing season and affecting quality and yield. Considerable effort has therefore been directed toward understanding how plants respond and adapt to low temperature. *Arabidopsis thaliana,* like many plants, increases its freezing tolerance when exposed to low nonfreezing temperatures (reviewed in [1]). This process of cold acclimation is a multigenic and quantitative trait that is associated with complex physiological and biochemical changes. These changes are extensive and affect growth and water balance, the accumulation of compatible solutes, membrane and cell wall composition, antioxidant production (increased), and cold-regulated (COR) gene expression and protein levels [1–3]. Traditional approaches have identified around 200 cold-responsive genes, but more recently this list has been extended by several hundred using expression profiling technologies [4–9]. Many of the expression changes can be related to the well-documented biochemical changes listed above, while others provide new information. The characterization of genes that respond to cold in *Arabidopsis* is important to understand the response of plants to low temperature and the processes involved in plant cold acclimation. Such information will help in the development of strategies for the improvement of freezing tolerance in crop plants.

The regulation of cold-responsive gene expression has received much attention and has been recently reviewed [3,10]. The *C-repeat binding factors (CBFs)-1, -2,* and *-3* (also known as *dehydration-responsive element binding1 (DREB1)-b, -c,* and *-a,* respectively), have been a focus of research, including the identification of target genes involved in the cold response [11,12]. The overexpression of the CBF genes has been shown to have large effects on the cold-responsive transcriptome and metabolome of *Arabidopsis,* and their activities may be functionally redundant [6,11,13]. However, analysis of a mutant in which the *CBF2* gene was disrupted revealed that this gene negatively regulates *CBF1* and *CBF3* expression [14]. Other transcription factors have been shown to act as positive [4] or negative [15] regulators of, or in addition to [16], the CBF pathway. Recently, ZAT12 was shown to down-regulate the expression of the CBF genes and to have a cold-responsive regulon that partially overlapped with *CBF2* [9]. Clearly, the transcriptional regulation of cold-responsive gene expression is complex. The identification of cold-responsive genes that are potentially under the control of these transcription factors will be useful to decipher their function and relative importance.

Despite the wealth of published data and the increasing availability of public datasets (e.g., NASCArrays, AtGenExpress), there is currently no consensus on the number and identity of cold-responsive genes in *Arabidopsis;* reports and estimates vary from less than 100 to about 1,000 [5–8]. This variation is related to the diversity of growth conditions and experimental treatments of the plants and of the profiling technologies used. In addition, the development of standards for microarray experiments and data analysis has left many of the initial studies lacking sufficient replication or a thorough statistical evaluation of the data. Moreover, in common with many other treatments that have large effects on gene expression, there has been little discussion of the assumptions underlying normalization for microarrays and the confirmation of observed changes. Finally, although results from expression profiling have been discussed in relation to previously reported cold-responsive genes, there has been no large-scale validation of the observed changes using an independent platform. The discussions of these results have focused mainly on individual or small groups of genes, and functional classifications have not been related to, or statistically tested against, those of all genes.

Here, we present replicated statistical comparisons from independent microarray platforms of the changes in gene expression after 14 d of cold acclimation. A comparison of these data with published and publicly available datasets allowed us to compile consensus lists of the most consistent changes for short- (12 h or less), medium- (24–48 h), and long-term (more than 48 h) cold-responsive genes in *Arabidopsis*. Using improved annotation, visualization, and statistical testing, we focused on global changes and patterns to provide new insights into which processes are most important during exposure to low temperature.

## Results

### Data Quality Assessment

As a necessary prerequisite for data interpretation, the first priority should be the assessment of data quality. This can be separated into two basic considerations: the technical quality of the arrays, and the validity of the expression estimates. The former approach is straightforward, with all our arrays passing both the manufacturers guidelines and additional data quality assessment using the bioconductor (http://www.bioconductor.org/) and R software. The second approach is often addressed by confirming a small number of candidate genes using RNA blots or quantitative or semi-quantitative RT-PCR with one or two housekeeping genes as controls. This is not practicable when confirmation of many changes is required. Therefore, we used two independent microarray platforms: the Affymetrix ATH1 genome array (subsequently referred to as ATH1) [17] and the MWG Biotech 25k *Arabidopsis* 50-mer oligonucleotide array (MWG).

**Figure 1:**
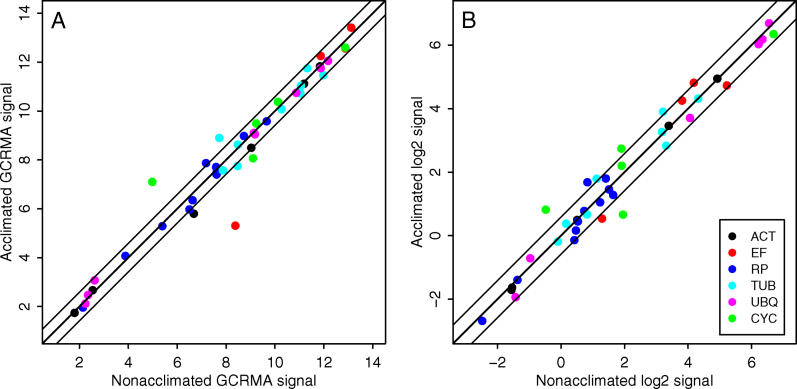
Comparison of Housekeeping Gene log2 Signal between Nonacclimated and 14 d Cold Acclimated *Arabidopsis* Data for the ATH1 (A) and MWG (B) arrays are shown. Genes annotated as actins (ACT), elongation factors (EF), DNA-dependent RNA polymerases (RP), tubulins (TUB), poly-ubiquitins (UBQ), and cytosolic cyclophilins (CYC) were used. Genes used are listed in Table S7.

The possibility of a global change in cellular mRNA abundance needs to be considered, as this may confound the interpretation of data from expression profiling due to bias introduced during normalization (see Discussion). To our knowledge, this has not been discussed in relation to plant studies. For both platforms, we compared signal intensities for a large number of common housekeeping genes across treatments. Additionally, we compared our results to changes reported using conventional non-microarray approaches. The data for housekeeping genes show good linear correlations (Figure 1). The mean absolute log2 fold change in gene expression during cold acclimation for the housekeeping genes on the ATH1 and MWG arrays are 0.35 and 0.41, respectively. These are lower than the 0.39 and 0.62 obtained using permutations of the same number of randomly selected genes of similar intensities. We compared our data to previous reports of genes responding to low temperatures in the long term (more than 48 h) that did not use profiling technologies. For genes mentioned in these reports and present on either of the arrays, signal changes matched for 15/16 and 15/17 down-regulated genes, and 23/29 and 26/29 up-regulated genes for the ATH1 and MWG arrays, respectively. These signal changes are significant in at least one dataset for 13/18 down-regulated and 16/29 up-regulated genes.

### Comparison of ATH1 and MWG Arrays

**Figure 2:**
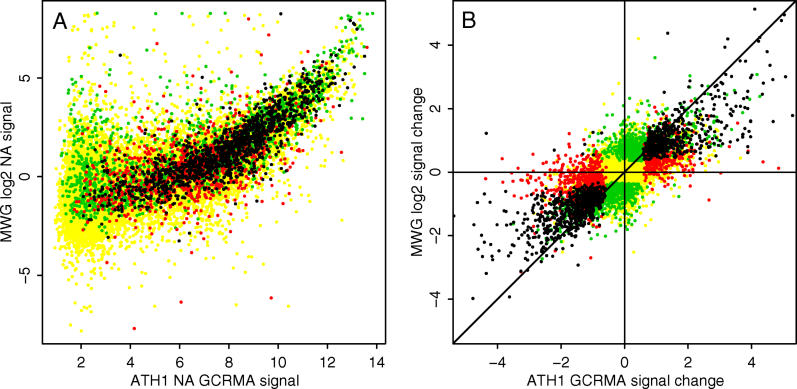
Comparison of log2 Signal and Signal Changes between the ATH1 and MWG Arrays Shown here is a comparison of log2 signal for nonacclimated plants (A) and log2 signal change during cold acclimation (B), between the ATH1 and MWG arrays. Data are for the 20,159 genes that are represented by probes on both the ATH1 and the MWG arrays. Genes are colored on the basis of their detection as significantly (FDR *p* < 0.05) differentially expressed in response to 14 d cold acclimation using the two-array datasets. Black, both datasets; green, MWG; red, ATH1; yellow, neither.

Recently, there has been considerable interest in cross-platform reproducibility of profiling experiments [18]. This has mainly focused on animal studies, with only one plant study in which multiple array technologies were used to confirm expression changes [12]. Arrays can be compared on the basis of absolute expression or the change between treatments. Using data for all 20,159 AGI locus codes that are represented on both arrays, there is a correlation of 0.7 for both nonacclimated log2 absolute expression and log2 change during cold acclimation. However, this gives a distorted view of the reproducibility of these parameters due to differences in the distribution of their values. Gene expression changes during cold acclimation are clustered around zero but extend as a linear distribution, while the log2 nonacclimated expression comparison is linear only at higher intensities (Figure 2). At low intensities, the MWG expression estimates are more compressed than those from the ATH1 array.

It is now becoming accepted that *p*-values from statistical testing are better criteria for defining differential expression than arbitrary fold-change cutoffs. However, the validity of *p*-values arising from statistical tests of differential expression has been the subject of debate, and it is necessary to discuss this issue before interpreting results based on these values. The distribution of unadjusted *p*-values resulting from a test should show an expected distribution. This distribution can be expressed as a mixed model of the *p*-values arising from the null hypothesis, which are distributed uniformly on the interval (0,1), and the *p*-values of the alternative hypothesis, which have a higher density of small *p*-values [19]. The *p*-values from the test for differential expression using the MWG data fit this model perfectly, and those from the ATH1 data fit very closely (very slight overabundance of high *p*-values). This indicates that the chosen test is valid and that a model can be used to estimate true and false negatives and positives [19]. We used a linear step-up false discovery rate (FDR) correction of *p*-values for multiple testing [20], which has been shown to provide conservative FDR control for microarray data [21]. An FDR-corrected *p*-value of less than 0.05 means that less than 5% of the total number of significant values (genes) below this threshold are false positives. This differs from the uncorrected *p*-value, which gives the proportion of all tests (total genes on the array) that falsely declare a significant difference.

**Figure 3:**
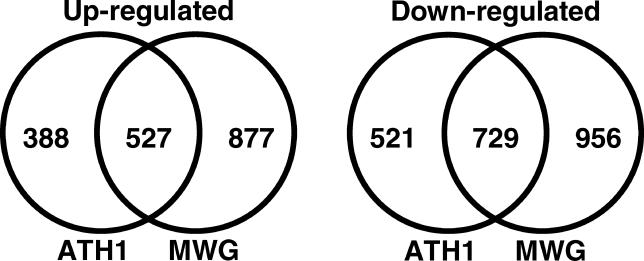
Comparison of Significant Expression Changes between the ATH1 and MWG Arrays Venn diagrams showing the overlap between genes detected as significantly (FDR *p* < 0.05) up- or down-regulated using data from the ATH1 and the MWG arrays. Only data for the 20,159 genes that are represented by probes on both arrays are shown.

The MWG data analysis identifies more genes that are differentially expressed in response to 14 d of cold treatment than the ATH1 data analysis at any given significance threshold. This effect is greater than can be explained by the larger size of the MWG array. At the FDR *p*-value of less than 0.05, approximately 900 more genes are significantly regulated according to the MWG data than the ATH1 data. This is most likely related to differences in the statistical analysis of the data necessitated by the technical replication of the MWG array hybridizations. Among genes present on both arrays, the ATH1 data reveal 2,165 that change significantly at the FDR threshold of 0.05, of which 58% are also significant at the same threshold using the MWG data (Figure 3). Of the 1,263 genes that have an FDR *p*-value of less than 0.05 for both arrays, only seven have opposite ratios (see Figure 2B). As there are high false-negative rates associated with these *p*-value thresholds (see below), it is not sufficient only to compare numbers of significantly changed gene expression levels at a given threshold. The overall consistency of the data is evident from the fact that only 6.5% of the 2,643 genes showing a significant change in expression on the ATH1 arrays show an opposite direction of change using the MWG arrays (see Figure 2B). Of these 141 genes, the majority (64%) have FDR corrected *p*-values greater than 0.5 in the MWG data, indicating that these ratios are not significant. In contrast, the FDR *p*-values of the remaining genes, which agree in direction between the two platforms, are heavily biased toward the MWG data also giving a low FDR *p*-value.

### Comparison with Previous Studies

We have already shown the consistency of our data in comparison to previous studies using nonprofiling techniques. As we have compiled a database containing the expression changes for cold-responsive genes from other expression profiling experiments, we can easily compare between studies. Studies that had multiple observations for a single time class were averaged. As different data analysis methods were used, and for simplicity, we express the similarity between two studies as the proportion of the common differentially expressed genes that agree in their direction of change. Comparisons between all studies indicate that the main effects leading to higher agreement are the duration of the cold exposure, the array type, and the replication/data analysis (Table S1). ATH1 data analyzed using the Microarray Suite 5 (MAS5) software form the most obvious group. Datasets that have only one biological and/or technical replication and lower cutoffs have lower agreement. The classification of the studies according to time classes of short, medium, and long term is supported by the comparisons between the studies. The minimum agreement between our data and other long-term studies was 0.83 and 0.76 for the ATH1 and MWG datasets, respectively, obtained in comparison to Vogel-P 7d (Table S1). There was lower agreement with the medium-term datasets, and agreement was lowest in comparison to the short-term datasets.

### Cold-Responsive Gene Expression

There have been previous estimates of how many genes respond to low temperature, but these have tended to be conservative. In the long term, with an FDR *p*-value threshold of less than 0.05, we detect 2,297 and 3,379 cold-responsive genes using the ATH1 and MWG arrays, respectively. These are underestimates due to the high false-negative rates. If we model the *p*-value distributions [19] using either of the datasets, we find the true positive rate is likely to be in excess of 10,000 genes. In the short and medium term, there are no suitable datasets to estimate the true number of cold-responsive genes. However, we made comparisons using available data to obtain an approximation of the relative numbers of cold-responsive genes. These comparisons indicate that at 24 h there are either more genes changing or that the magnitude of changes is greater in comparison to the long term. Of these changes visible at 24 h, it is likely that 20%–30% are seen within 3 h of cold treatment, and this could have increased to 75% after 12 h.

**Figure 4:**
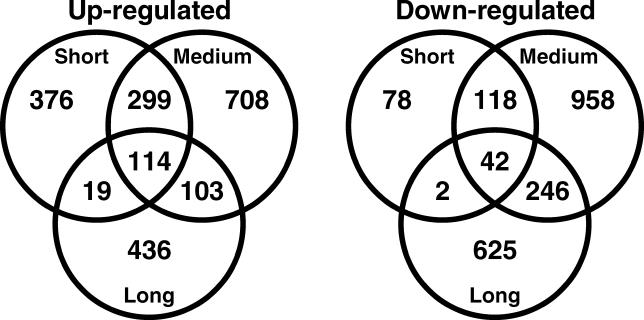
Comparison between Short-, Medium-, and Long-Term Cold Responsive Genes Venn diagrams showing the overlap between genes classified as being up- or down-regulated in response to short-, medium-, or long-term cold treatments. Full details are given in Materials and Methods and Table S2.

Obviously, some of these changes depend on the treatment and technology used, with the changes of most interest being those that are consistent across independent studies or technologies. We have used the available data to generate lists of the genes most consistently responding to low temperature in the short, medium, or long term (Table S2). Genes mentioned in these lists have been reported in at least two biologically and/or technically independent experiments. Specifically, for each independent study within a time class a gene was given a score of +1 for up-regulation and −1 for down-regulation. Those genes scoring 2 or higher or −2 or below were defined as cold responsive. These criteria allowed us to define 808, 1,224, and 672 genes as consistently up-regulated, and 240, 1,364, and 915 genes as down-regulated in response to cold in the short, medium, and long term, respectively (Figure 4). Of the up-regulated genes, 114 are common to all lists, while the number of common down-regulated genes is 42. Of the short-term cold responsive genes, about 55% and 17% are similarly changed in the medium and long term, respectively. Approximately 30% of the long-term cold-responsive genes are similarly changed in the medium term.

### Annotation-Based Analysis Reveals Key Changes for Cold-Responsive Gene Expression

It is clearly not possible to describe all changes reported for cold-responsive gene expression, and the reader is encouraged to explore the data using the Mapman plots [22,23] (S1–11, M1–11, and L1–11 in Dataset S1), the analysis of Mapman functional groups (Table 1), and that of The *Arabidopsis* Information Resource (TAIR) AraCyc pathways (Table S3). Our analysis did not focus on individual genes, as these can be identified directly (S1–11, M1–11, and L1–11 in Dataset S1). We also did not directly compare the number of up- or down-regulated genes, as the differences in the numbers of regulated genes (Figure 4) would make some of the analysis trivial. Instead, we took a global view, where distributions of cold-responsive genes among different functional groups were tested for significant deviation from that expected for all genes within the database. Mapman functional groups and AraCyc pathways that were significantly under- or overrepresented in this analysis were identified using Fisher exact tests. Using the Mapman functional groups, unknown genes were significantly underrepresented among many of the gene lists (Table 1). The statistical analysis was repeated with these genes excluded, as their underrepresentation could increase the proportion of genes in functionally defined groups without pertaining to biological effects. The highlighting of *p*-values in Table 1 is based on their meeting a given cutoff in both analyses.

**Table 1:**
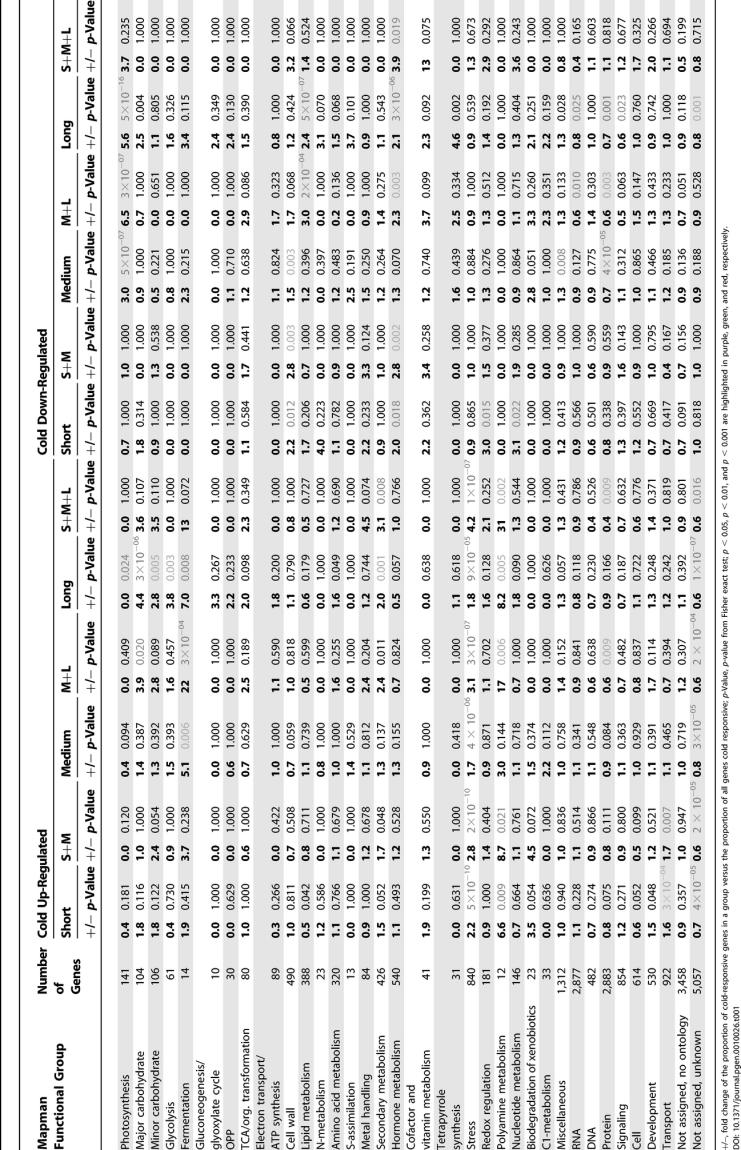
Statistical Analysis Showing Over- and Under-Representation of Mapman Functional Groups

+/−, fold change of the proportion of cold-responsive genes in a group versus the proportion of all genes cold responsive; *p*-Value, *p*-value from Fisher exact test; *p* < 0.05, *p* < 0.01, and *p* < 0.001 are highlighted in purple, green, and red, respectively.

The most affected group is that of stress, which is significantly (*p* < 0.001) overrepresented among cold up-regulated genes at all times (Table 1). Stress related genes up-regulated include those known to respond to temperature, drought, and salt (S1, M1, and L1 in Dataset S1). Other groups that have significantly more up-regulated genes than expected at most times are polyamine and secondary metabolism. The AraCyc pathway analysis reveals that flavonoid, lignin, phytoalexin, and suberin biosynthesis, and the initial phenylpropanoid reactions, which share many common genes, are overrepresented in the short to medium term (Table S3). Mapman visualization indicates that the up-regulated genes of secondary metabolism are involved mainly in the metabolism of dihydroflavonols, chalcones, and anthocyanins (S2, M2, and L2 in Dataset S1). The hormone functional group is the only one with significantly more down-regulated genes than expected at most times. This is mainly due to the down-regulation of auxin-induced or -responsive genes, although genes from the jasmonic acid and ethylene groups are also abundant (S3, M3, and L3 in Dataset S1). In the long term, signaling/response to gibberellin also has many down-regulated members. The only hormone biosynthetic pathway significantly down-regulated is brassinosteroid biosynthesis in the short and long term (Table S3A).

Short-term–specific responses are the significant overrepresentation of transport among up-regulated genes, and of nucleotide metabolism and redox regulation among down-regulated genes. The AraCyc pathway analysis also identifies purine biosynthesis from nucleotide metabolism as significantly overrepresented among down-regulated genes (Table S3A). In addition, the cell wall group is significantly overrepresented among down-regulated genes in the short to medium term (Table 1). Up-regulated genes involved in transport are thought to have roles as ATP binding cassette, sugar, and phosphate transporters and ATPases (S4 in Dataset S1). Changes in the transport group are generally similar in the medium to long term, although sugar transporters become predominantly down-regulated (M4 and L4 in Dataset S1).

In the short term, it may be expected that many of the observed changes are involved in the signaling and regulation of the responses to low temperature. However, the proportion of these genes up- or down-regulated is not significantly different from that expected (Table 1). Genes that do change are those involved in calcium, G-protein, and light signaling and those annotated as receptor or mitogen-activated protein kinases (S3 in Dataset S1). The significant overrepresentation of redox regulation among down-regulated genes indicates a possible importance in the initial response to low temperature.

The statistical analyses reveal that there are no functional groups that are changed specifically in the medium term (Table 1). The miscellaneous group contains significantly more down-regulated genes than expected, but the *p*-values indicate this is a medium- to long-term response. The peroxidase subgroup appears to be the most affected within this group, with eight of 72 members down-regulated, while in the long term group, ten of the 82 GDSL-motif lipases are down-regulated (M5 in Dataset S1). The other group that is overrepresented among down-regulated genes in the medium to long term is photosynthesis (Table 1). The down-regulation of photosynthesis is general, with the light reactions, Calvin cycle, and photorespiration all down-regulated in the medium to long term. Tetrapyrrole synthesis is significantly (*p* < 0.01) overrepresented among down-regulated genes in the long term (M6, L6 in Database S1). Genes from fermentation are up-regulated in both the medium and long term, including *lactate dehydrogenase, pyruvate decarboxylase,* and *alcohol dehydrogenase*.

The highest number of significantly affected groups is seen in the long term. In addition to the shared responses already listed, there are a number of long-term–specific changes. Primary metabolism is strongly affected, with the functional groups of minor and major carbohydrate metabolism and glycolysis all containing significantly more up-regulated genes than expected (Table 1). In addition, there could be a similar response for the tricarboxylic acid cycle (TCA) and amino acid groups, which also have low *p*-values. Major carbohydrate and lipid metabolism are significantly overrepresented among down-regulated genes. Many of these long-term changes are already observed in the medium term, as indicated by the proportion changes of the medium-term genes and the proportion changes and *p*-values of the genes that are common to both the medium and long term (Table 1). Sucrose synthesis and the conversion of UDP-glucose are both significantly overrepresented among long-term up-regulated genes, while starch degradation is significantly (*p* < 0.01) overrepresented among down-regulated genes (Table S3A). The functional groups of RNA, protein, and signaling all contain significantly fewer down-regulated genes than expected (Table 1).

### Analysis of COR Genes

It has been previously reported that many COR/late embryogenesis abundant (LEA) genes are cold induced [6]. These genes encode hydrophilic proteins that are thought to be important for freezing tolerance [3]. The hydrophilic nature of proteins encoded by cold-responsive genes of unknown function was used to designate them as putative COR genes [6]. An earlier comparative study on water stress in prokaryotes and eukaryotes had defined a subgroup of LEA proteins (including two COR proteins), called hydrophilins, that had a mean hydrophilicity of over 1 and a glycine content of over 0.08 [24]. Another group of LEA proteins, with hydrophilicity indices between 0 and 0.5, were not included in this classification.

Genes encoding highly hydrophilic proteins (hydrophilicity of about 1) are significantly more abundant than expected among short-, medium-, and long-term cold up-regulated genes in comparison to all genes (Table S4). This increased abundance is higher for genes that are up-regulated at multiple times. In contrast, genes down-regulated in response to cold encode proteins with hydrophilicity indices more similar to those expected by chance. Among long-term down-regulated genes, there are actually significantly fewer genes coding for highly hydrophilic proteins than expected (Table S4).

Many of the COR proteins have glycine contents of more than about 0.08 (Table 2). The hydrophilicity indices of these proteins range from 0.29 to 1.19 (Table 2). The proportions of genes coding for proteins with glycine content of more than 0.08 among those up- and down-regulated in response to cold are significantly higher than expected (Table S4). Among up-regulated genes there are many that belong to the abiotic stress group that contribute to this increase, while for down-regulated genes the increase comes from proteins in the photosynthesis, cell wall, and hormone functional groups. High-glycine proteins with roles in abiotic stress are mainly hydrophilic, with the notable exceptions of the highly hydrophobic RCI2A and RCI2B, known to be induced by abiotic stress [25]. Among hydrophobic proteins, enzymes of primary metabolism and transport proteins also contribute to the increased abundance of proteins with high glycine content. The genes up-regulated by cold include 58 encoding unknown proteins with high glycine content, 11 of which are induced at multiple times (Table S5).

**Table 2:**
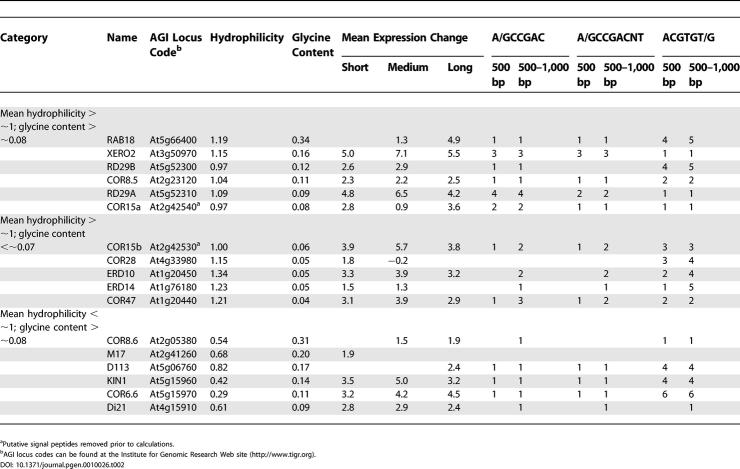
Mean Hydrophilicity, Glycine Content, Expression Changes, and the Frequencies of Cis-Elements for Known COR Proteins

^a^Putative signal peptides removed prior to calculations.

^b^AGI locus codes can be found at the Institute for Genomic Research Web site (http://www.tigr.org).

The predicted proteins from 12 short-, 20 medium-, and 13 long-term cold up-regulated genes meet the criteria of being hydrophilins. The proportions of these genes that are up-regulated are significantly higher than expected at all times, but highest in the medium to long term (Table S4). The majority of these proteins that are annotated interact with RNA, with roles as transcription factors, splicing factors, and ribosomal proteins. The COR proteins RAB18, XERO2, RD29A, RD29B, COR15a, and COR8.5 [6] are also hydrophilins (Table 2). There are 11 up-regulated genes encoding unknown proteins that are possible hydrophilins, most of which have not been previously reported (Table S5). In addition, another unreported protein meeting the hydrophilin criteria is annotated as an abscisic acid (ABA)-responsive protein similar to KIN1 from *Brassica napus*.

### Transcriptional Regulation

Transcriptional regulation of the response to low temperature is the subject of intensive research. Our analysis focuses on identifying families that are overrepresented among cold-responsive transcription factors. These families may be important during cold acclimation. The complexity of the overall regulation is clear, with 111, 141, and 58 known and putative transcription factors up-regulated, and 25, 133, and 84 down-regulated in the short, medium, and long term, respectively. In the short term there is a significant (*p* < 1 × 10^−5^) overrepresentation of the APETALA2/ethylene response element binding protein (AP2/EREBP) family among cold up-regulated transcription factors (Table S6). This family contains 17 up-regulated members, including *CBF1–CBF3, DREB2B,* and *RAV1* (S7 in Dataset S1). Although many members continue to be up-regulated, the total number is not significantly different to that expected in the medium term and less significant in the long term. The *CONSTANS* (CO)-like family is also significantly (*p* < 1 × 10^−6^) overrepresented among short-term up-regulated transcription factors, but this overrepresentation continues to be significant (*p* < 1 × 10^−3^) in the medium to long term (Table S6). In the medium term the type B response regulator, basic leucine zipper, heat shock transcription factor (HSF), and TCP-domain families are significantly overrepresented among up-regulated transcription factors. The *p*-values indicate that the HSF and TCP-domain families are also overrepresented in the short term. In the long term the auxin response factor (ARF) family is also likely to be overrepresented (*p* = 0.051) (Table S6). There are 13 transcription factor genes that are up-regulated at all times, and these include the AP2/EREBP members *RAP2.1, RAV1,* and *CBF2*, and four from the CO-like family (S7, M7, and L7 in Dataset S1). There are too few short-term down-regulated transcription factors to draw any conclusions. In the medium term there is a significant overrepresentation of members from the MYB-related and Aux/IAA (*p* = 0.057) families among down-regulated transcription factors (Table S6). In the long term, the basic helix-loop-helix (bHLH) and Aux/IAA families are significantly overrepresented (M7 and L7 in Dataset S1).

### Promoter Analysis of Cold-Responsive Genes

The CBF/DREB transcription factors specifically bind the dehydration-responsive element (DRE)/C-repeat cis-acting element that is present in the promoter regions of many COR genes [26,27]. The DRE core motif is A/GCCGAC, although recently the consensus motif for the binding of CBF3/DREB1A has been further characterized as A/GCCGACNT [12]. This study also found the ABA response element (ABRE) ACGTGG/T to be overrepresented in the promoters of cold up-regulated genes. We compared the frequencies of the DRE core, the A/GCCGACNT motif, and the ABRE motif in the 500- and 500–1,000-bp promoter regions of our consensus cold-responsive genes. Statistical comparisons were made against the frequencies found for all genes in our database. The ABRE motif was significantly (*p* < 1 × 10^−6^) overrepresented in the 500-bp promoter regions among all cold up-regulated genes, but was significant only in the short term for motifs found in the 500–1,000-bp regions (Table 3). The A/GCCGACNT motif was significantly (*p* < 1 × 10^−8^) overrepresented in the 500-bp promoter regions among all cold up-regulated genes (Table 3). In addition, the A/GCCGAC and A/GCCGACNT motifs were generally overrepresented in the 500-bp and 500–1,000-bp promoter regions, respectively, although these differences were only significant at the *p* < 0.01 level for medium-term up-regulated genes (Table 3). The increase in the frequency of DRE elements is greatest for the A/GCCGACNT motif, and increases further among genes induced at multiple times (Table 3). Of the genes up-regulated in the short, medium, and long term we identified, respectively, 80, 118, and 63 genes (165 unique) containing the A/GCCGACNT motif within their 500-bp promoter regions. There were 83 additional genes with the A/GCCGACNT motif within 500–1,000 bp upstream and 230 additional genes with the A/GCCGAC motif within 500 bp upstream of their start codons. In total, 478 of the 2,055 up-regulated genes meet these criteria and are therefore potential targets for CBF transcription factors. This gives a proportion of 0.23, which is significantly higher than the 0.16 found among all genes in our database. However, our statistical analysis revealed that the A/GCCGACNT motif within the 500-bp promoter region is the most specific to cold-responsive genes, occurring in the promoters of twice the number of expected genes.

**Table 3:**
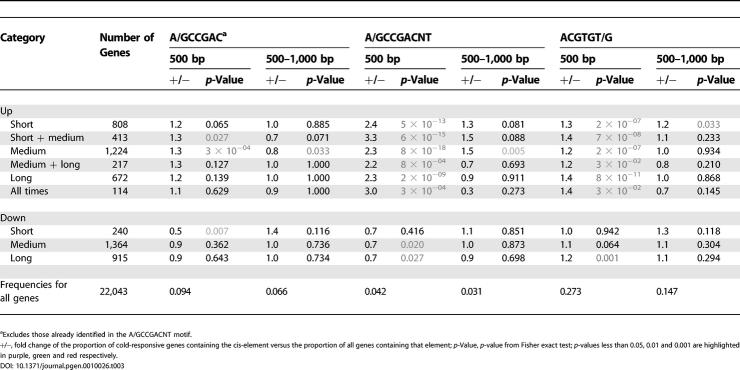
Enrichment of DRE and ABRE Cis-Elements among Consensus Cold-Responsive Genes

^a^Excludes those already identified in the A/GCCGACNT motif.

+/−, fold change of the proportion of cold-responsive genes containing the cis-element versus the proportion of all genes containing that element; *p-*Value, *p*-value from Fisher exact test; *p*-values less than 0.05, 0.01 and 0.001 are highlighted in purple, green and red respectively.

## Discussion

There are now a large number of studies investigating the changes in gene expression in response to low temperature (for example, see the Web sites at http://affymetrix.arabidopsis.info/ and http://www.arabidopsis.org/info/expression/ATGenExpress.jsp) [4–9]. Although some of these studies have used time courses, for individual time points biological variability has been sampled only in duplicate at most. This has led to the use of arbitrary cutoffs to define differentially expressed genes. In addition, only small numbers of candidate genes have been confirmed by independent means. In this study, we report a genome-wide validation of expression changes using independent microarray platforms for *Arabidopsis,* and through statistical analysis we used these data to determine changes in gene expression after 14 d of cold acclimation. We integrated these data with comprehensive analyses of published and publicly available data to define consensus lists of short-, medium-, and long-term cold-responsive genes.

### The Analysis and Validation of Expression Profiling Data

Despite the large number of expression profiling studies on plants, there is often little discussion of the data analysis and validity of the measured expression estimates. In almost all microarray studies there is the usually unmentioned assumption that either the vast majority of transcripts do not change, or that any changes that do occur are balanced [28]. This is because the expression levels of all genes are used for normalization. Even the most basic normalizations, such as a mean or median scaling, are based on the assumption that the average numbers and abundances of mRNAs are similar between samples. Other normalizations, such as quantile or loess, additionally assume similar distributions of mRNA abundance by fitting a linear relationship between all samples. This means that the expression estimate of a single mRNA should be considered as relative to the total combined expression of all transcripts (i.e., total mRNA). The assumption that most genes do not change is probably violated in many studies, but is often not evident, because stringent but arbitrary cutoffs are used that reveal relatively few changes. If the majority of changes occur in one direction, or for transcripts of a certain intensity (e.g., highly expressed genes), then the total amount or distribution of mRNA will change and any internal normalization will lead to bias due to the reasons highlighted above. As normalization is necessary to remove technical variability, and without it most patterns would be obscured by noise, these issues must be considered before data can be confidently interpreted.

To investigate the possibility of bias due to normalization, we compared the expression of a large number of commonly used housekeeping genes between treatments. Genes annotated as tubulin, actin, polyubiquitin, DNA-dependent RNA polymerase, cytosolic cyclophilins, and elongation factors were selected. It seems likely that if there were changes in the total amount or distribution of mRNA, then diverse housekeeping genes such as these would show a bias in their correlation between treatments. Similar to previous reports [7,29], there are effects on the expression of individual genes but overall there is a close correlation, giving no indication that any global mRNA changes occurred (see Figure 1). Of 44 selected housekeeping genes, only two are changed in both of our array datasets, indicating only slight changes in gene expression. Additionally, our data show 93% agreement with long-term cold-responsive genes reported using nonprofiling techniques (see Table S1). Together, these approaches offer good evidence that global mRNA changes did not occur in our study.

Previous studies in the field of cold-responsive gene expression have not considered global mRNA changes. Using our database of cold-responsive genes, we looked at the expression of the selected housekeeping genes across all studies (Table S7). In the long term, there are the two up-regulated genes from our study, plus one additional down-regulated gene. In the short term there is just one up-regulated gene. However, in the medium term there are no up-regulated genes, but six of the 44 genes are down-regulated. This has a high probability of not being due to chance (*p* = 0.052), which could be due to a number of reasons. First, the two elongation factors, three DNA-dependent RNA polymerases, and *Actin 11* could be cold-responsive isoforms of these gene families. Second, the high expression of housekeeping genes means that they are more likely to be detected as “present” using the Affymetrix MAS5 software, and as only genes “present” before cold treatment can be down-regulated, this would contribute to their increased abundance compared to all genes. Finally, the possibility of a global change in total mRNA needs to be considered. In the AtGenExpress time series, there are about 9-, 3-, and 2-fold more up-regulated than down-regulated genes at 1, 3, and 6 h respectively, while at 12 h these changes are balanced (see Table S2). If a sufficient proportion of transcripts increased in abundance, an increase in the total amount of mRNA within the cell could occur, and, following normalization, genes that are not changed could be detected as down-regulated. However, the high magnitude of the housekeeping genes down-regulation, and the low proportion of early unbalanced changes, indicate that the first two reasons are the more likely.

### Comparison of ATH1 and MWG Arrays

The agreement between expression changes identified using independent *Arabidopsis* microarray platforms has not received much attention. This leaves some doubt over the universal validity of results obtained using a single technology, even if several of the larger changes were confirmed by independent methods. In our study, we find a good correlation between the results obtained using the ATH1 and MWG arrays. Although transcripts with higher abundances should give higher intensities, differences in probe sequence could lead to discrepancies. This can be due to the binding efficiency of the probe to the target (G/C content and interference from labeled nucleotides) and the labeling potential of the target sequence. Considering these factors, the correlation of the actual intensities between the two technologies is better than expected (see Figure 2A). This comparison of absolute expression values suggests that the ATH1 array has a greater dynamic range at lower intensities, while the arrays behave more similarly at higher intensities. This could reflect the decreasing influence of the mismatch probes at higher intensities. To increase dynamic range, MWG combines measurements using different photomultiplier tube gain settings, and this may also contribute to the nonlinear relationship. The correlation of 0.7 between absolute signal for the MWG and ATH1 arrays is very similar to the recent report of 0.72 between the Agilent 60-mer oligonucleotide and ATH1 arrays [30]. However, this correlation was obtained for filtered data and no details of the distribution were given.

If statistical testing is used to detect differential gene expression, it is necessary to assess the suitability of the chosen test. The unmodified t-statistic can lead to false positives from genes with low variances but very small expression changes. Various methods have been proposed to overcome this limitation [31]. One of the most recent and versatile developments, used in the present study, is the empirical Bayes moderation of t-statistics implemented in the Limma Bioconductor package [32]. Once a statistical test is used, the validity of the *p*-values and their correction for multiple testing at an accurate threshold requires them to fit an expected distribution [19]. The ATH1 and MWG data both meet these criteria, allowing us to compare significant changes in expression across the two platforms.

It would be expected that the signal changes are more stable between technologies than are absolute expression levels, as sequence-specific interference will have less effect. Signal changes show a linear correlation between both array datasets, although the magnitudes of changes are smaller for the MWG than the ATH1 data (see Figure 2B). As with the absolute expression, this could indicate a lower dynamic range for the MWG array. Alternatively, the linear correlation could indicate a difference in scale between the two expression measures. Keeping these small differences in mind, there is close agreement between the significant changes detected by the two technologies (see Figures 2B and 3), and this agreement would likely be higher were it not for the high numbers of false negatives using both technologies. The low proportion of opposite changes and the general agreement of *p*-values also indicate close agreement. Taking these factors into consideration, it seems likely that the vast majority of significant changes are due to biological rather than technical variation, and that data from either microarray can be interpreted with confidence. Similar conclusions have been made recently for the comparison of mammalian Affymetrix and spotted oligonucleotide arrays [18,33].

### Comparison of Previous Studies

All of the comparisons made between previous studies were based on genes that changed in both studies. The proportion of all changes that were common to both studies was extremely variable (see Table S1B). The impact of the high false-negative rates on comparisons has already been discussed. The different cutoffs used in previous studies are likely to lead to increased false-negative rates. Therefore, the massive effect of using cutoffs on the change/no change classification of a gene, and the different genes represented on each array, allow no interpretation of the proportion of common genes. These factors have little effect on the comparison of expression changes for genes that are identified in both studies, so this represents the best method of comparison. In general, considering the diverse nature of growing conditions, treatments, and profiling technologies, the agreement between all studies was good. The close agreement between the ATH1 datasets is obviously due to the use of the same array, normalization, and analysis. The agreements within short-, medium-, and long-term data support our separation of studies into these three classes. Across all time classes, the lowest agreement with our datasets was found in comparison to poorly replicated studies using lower cutoffs. The use of a single biological sample hybridized to either one or multiple chips or duplicate biological samples hybridized to a single chip will result in an increased false-positive rate caused by the underestimation of biological or technical variability.

### Cold-Responsive Gene Expression

In studies using the approximately 8,000-gene *Arabidopsis* Genome Array (AtGenome) array, the numbers of genes reported to respond to low temperature are about 300 [6] and 1,100 [7]. These data suggest that 4%–14% of the *Arabidopsis* genome is cold responsive. These studies focused on controlling the false-positive rate and, with no possibility of statistical analysis, there was little discussion of false negatives. Our data indicate that the true number of cold-responsive transcripts is likely to be approximately 45% of those represented on the array. A comparison of the variability of all datasets indicates that, at 24 h, either more genes change or the magnitude of changes is greater than for other times, and that of these genes, significant proportions change in the short term. Such large numbers of cold-responsive genes are clearly higher than most expectations. These estimates will include even those genes that show small changes in expression. Small changes of gene expression of 10%–50%, although generally not considered, could be important for many responses, including that to low temperature. These changes may be more significant if they are coordinated within functional groups or specific pathways. Improved gene ontology and visualization of significant changes will be important if so many changes are to be meaningfully interpreted.

### Integration of Data from Diverse Experiments into a Single Database

In the present study, samples harvested after 14 d of cold acclimation were used to reveal the stable changes in gene expression required to maintain the cold-acclimated state. This is before any new leaves develop at 4 °C. We chose to use soil-grown plants grown under a photoperiod, as these conditions are more similar to natural conditions. Obviously, under any experimental design, not all changes are relevant to cold acclimation, as many could be specific to the conditions used. Genes that are consistently cold responsive under diverse experimental conditions are likely to be of more general importance in the response of plants to low temperature. Therefore, we have incorporated all available data into consensus lists of short-, medium-, and long-term cold-responsive genes. These include data from plants of different ages grown on agar plates, in hydroponics and soil, and under continuous light or different photoperiods.

We identified 808, 1,224, and 672 up-regulated genes, and 240, 1,364, and 915 down-regulated genes in response to cold in the short, medium, and long term, respectively (Figure 4). These consensus lists offer the most comprehensive overview of cold-responsive gene expression presently available, to our knowledge. Moreover, the complete database offers an “at-a-glance” summary of the cold responsiveness of 22,043 genes (see Table S2). This will be a valuable tool for single- and multi-gene characterizations.

### Annotation Based Analysis Reveals the Most Prevalent Cold Responsive Expression Changes

The Mapman software offers improved ontology and the ability to visualize gene expression overlaid onto biochemical pathways or diagrams [22,23]. In addition, the TAIR AraCyc pathways detail genes involved in various biological processes. The complete datasets are available in Dataset S1 and Table S3, allowing the reader to obtain detailed overviews of cold-responsive gene expression. As there have been previous studies on the changes in gene expression in response to cold, many of the observed changes have been discussed [5–8]. These studies have mainly focused on individual or small groups of changes. Moreover, discussion of functional classification has not been related to, or statistically tested against, that of all genes. The knowledge that the number of genes changing expression within a functional group is significantly different from that expected by chance is important information that has been largely neglected in plant studies.

The overrepresentation of the stress group among up-regulated genes is expected and clearly related to the literature reports incorporated into our database that have also been used to provide gene ontology. The up-regulation of genes involved in temperature, drought, and salt stresses is known and reflects the cross-talk between signaling pathways [8]. Among short-term up-regulated genes there is a significant overrepresentation of transport proteins, including ATP binding cassette, sugar and phosphate transporters, and ATPases, at the plasma, vacuolar, and plastid membranes. This indicates that, in addition to changes in their metabolism, the transport of these substances into, out of, and within the cell may be an essential component of the initial response to low temperature. The transport of sugars appears to be a short-term–specific response, with the majority of sugar transporters becoming down-regulated in the long term. The redistribution of sugars may become less important as rearrangement of carbohydrate metabolism becomes established. The significant overrepresentation of carbohydrate metabolism among long-term up-regulated genes asserts that such rearrangement is occurring.

The accumulation of many compatible solutes and metabolites is known to occur in response to low temperature [13,34,35]. The well-documented increases in proline, and raffinose involve the increased expression of the *P5CS2* and *AtGolS3* genes, respectively [13]. The increased abundance of enzymes responsible for raffinose synthesis may be more general, as in addition to *AtGolS3,* genes encoding three other galactinol synthases (Institute for Genomic Research [http://www.tigr.org] Arabidopsis Genome Initiative [AGI] locus codes At1g56600, At2g47180, At3g28340), a raffinose synthase (At5g40390) [36] and enzymes responsible for myo-inositol and sucrose synthesis are all up-regulated in response to cold. The recently reported increases in pyruvate, alpha-oxoglutarate, succinate, and fumarate [13,34], are accompanied by increased expression of at least one enzyme responsible for their synthesis in the medium or long term, indicating a general increase in TCA cycle activity. The discussed overrepresentation of enzymes of carbohydrate metabolism includes also those involved in the production of glucose-6-phosphate, fructose-6-phosphate, glucose, maltose, fructose, sucrose, and trehalose, all of which accumulate in response to cold [13,34]. Expression changes can also explain many other metabolite changes, but caution is needed, because so many metabolite and expression changes occur, that patterns can always be found. Good annotation and statistical testing provide a more robust basis for conclusions based on global changes of pathways or functional groups. The Mapman and AraCyc analyses reveal coordinated changes within primary metabolism (S8–S10, M8–M10, and L8–L10 in Dataset S1). In the short term, genes encoding enzymes for sucrose and starch degradation are up-regulated, while in the medium to long term, sucrose degradation by invertase and starch degradation become down-regulated [35]. Sucrose degradation by sucrose synthase continues to be up-regulated, and enzymes for the conversion of the resulting UDP-glucose and fructose are also up-regulated. These could then enter into glycolysis and subsequently into the TCA cycle or fermentation, all of which are overrepresented among long-term up-regulated genes. This broad up-regulation of primary metabolism could explain the large number of metabolites that increase in response to cold acclimation [13,34].

There is a significant overrepresentation of down-regulated genes of lipid metabolism in the long term. Cellular membranes are a primary site of freezing damage, and changes in their composition are thought to be important for cold acclimation [1]. There is an increase in the proportion of di-unsaturated fatty acids which can reduce freezing-induced membrane damage in the plasma membrane [37]. The up-regulation of desaturases has been reported and these might be important in the conversion of existing fatty acids [38,39]. The down-regulated subgroups of lipid metabolism are those involved in fatty acid synthesis and elongation, synthesis of phospholipids and steroids/squalene, and degradation by lipases and lysophospholipases. Such a general reduction of lipid metabolism is probably related to lower demand due to the reduced growth at low temperatures. However, it could also indicate a key role for transcriptional down-regulation in changing enzyme activities necessary to maintain the altered lipid composition seen after cold acclimation [37]. Cell wall modification also occurs during cold acclimation [1] and, in common with lipid metabolism, our analysis shows that down-regulation may be important. In contrast, this down-regulation occurs in the short to medium term, indicating a possible role in the initial reduction in growth rate in response to low temperature.

Recent work on the response of carp to low temperature also identified genes encoding TCA cycle and mitochondrial electron transport components and transport as prominent functional groups among up-regulated genes [40], indicating possible functional similarities to *Arabidopsis*. These authors also made comparisons with earlier work on yeast, and although some genes such as delta-9-desaturase and translation initiation factors also have homologs up-regulated in *Arabidopsis*, the numbers are not sufficient to draw conclusions on conservation among functional groups.

### The Down-Regulation of Photosynthesis and Coordinated Changes in Other Pathways as Significant Response to Low Temperature

In *Arabidopsis,* 3 d of cold acclimation at 5 °C inhibited light-saturated rates of photosynthesis by approximately 75% [41]. The down-regulation of photosynthetic genes in response to cold has been previously reported [41,42], yet their significant overrepresentation among medium- to long-term down-regulated genes has not. These changes are massive, with 3- and 5-fold the expected number of genes down-regulated in the medium and long term, respectively. This appears to be a general response involving all aspects of photosynthesis. Tetrapyrrole synthesis is also significantly overrepresented among down-regulated genes. Light signaling contains higher proportions of down-regulated genes in the medium to long term (S3, M3, and L3 in Dataset S1), but in contrast, is mainly up-regulated in the short term, probably resulting from a transient “shock” prior to adaptation [6].

In cereals there is a short-term phosphate limitation of photosynthesis and a reduction in the supply of ATP, while in *Arabidopsis* ATP and ATP:ADP ratio actually increase [41]. However, in *Arabidopsis* it was concluded that low phosphate still plays an important role in triggering cold acclimation [42]. Such limitation is supported by the short-term down-regulation of a putative phosphate-induced gene (AGI locus code At4g08950). Our functional group and pathway analyses also revealed other potentially related changes during exposure to low temperature. The overrepresentation of transporters in the short-term includes ATPases and phosphate transporters which could be important in the response to short-term phosphate limitation. In nucleotide metabolism there are significantly more genes down-regulated in the short-term than expected indicating a possible role in changing cellular energy status in response to cold. The down-regulation of apyrase *APY2* and an adenylate kinase (AGI locus code At5g47840) could both contribute to increase the ATP:ADP ratio. The significant overrepresentation of up-regulated genes of secondary metabolism at all times is mainly due to those involved in the synthesis of flavonoids (S2, M2, and L2 in Dataset S1). Flavonoids are known to accumulate during plant stress and they are thought to protect cells from photo-oxidative stress by absorbing UV light [43]. This can prevent damage to photosystem II by increased excitation due to the reduced electron consumption at low temperatures [44].

As discussed, exposure to low-temperatures can lead to oxidative damage, particularly under high light, so reduced light intensities are generally used to minimize these effects. Most studies incorporated into the consensus lists of cold-responsive genes used reduced intensities, and as previously suggested [6], many of the changes of photosynthesis and related processes could represent general responses to low light conditions. Therefore, although the occurrence of these changes is not in doubt, their specificity and importance for plant cold acclimation remain to be fully established.

### COR/LEA Proteins and Their Importance for Plant Freezing Tolerance

One group of proteins that has received much attention is the COR/LEA proteins, some of which have protective effects during freezing [45,46]. In order to better characterize these proteins, we calculated mean hydrophilicity and glycine content of the proteins encoded by our consensus cold-responsive genes. The involvement of hydrophilic proteins in the response to cold [3] is supported, as hydrophilic proteins are significantly more abundant than expected among cold up-regulated genes. Of the established COR proteins, COR15a, COR15b, XERO2, RD29A, RD29B, RAB18, COR47, ERD10, and ERD14 have mean hydrophilicity greater than about 1 (see Table 2). Proteins with high glycine content are also significantly more abundant among cold-responsive genes. However, this increased abundance is due only to genes from the stress functional group for up-regulated genes. The COR proteins XERO2, RD29A, RD29B, RAB18, D113, M17, COR15a, COR15b, Di21, KIN1, and COR6.6 all have glycine contents of over about 0.08 (see Table 2). The hydrophobic cold-responsive proteins RCl2A and RCl2B [25] also have high glycine content. In fact, of the established COR proteins, only COR47, ERD10, and ERD14 have glycine contents of less than 0.08, and these proteins are all very hydrophilic with mean hydrophilicity greater than 1.2 (see Table 2). The cold-responsive genes encoding high-glycine proteins include 58 of unknown function, many of which may be similar to those already described (see Table S5).

The COR/LEA classification is broad, and their importance is often unclear. In order to improve their classification, we calculated the mean hydrophilicity and glycine content for COR/LEA proteins from various species shown to have effects that could relate to improved freezing tolerance (Table 4). COR15a has been shown to have protective effects on membranes during freezing [46], and conformational changes of maize DHN1 during binding to vesicles also suggest a role in the stabilization of membranes [47]. WCS120 from wheat and COR85 from spinach have been shown to increase enzyme stability during freezing [48,49], and PsLEAm stabilizes enzymes during drying [50]. Also, during drying a D-7 LEA protein was shown to stabilize sucrose glasses in vitro [51]. At the whole plant level, the overexpression of *HVA1* was shown to improve salt and drought tolerance in rice [52], while in *Arabidopsis* the overexpression of the dehydrins *RAB18* and *COR47* or *ERD10* and *XERO2* enhanced freezing tolerance [45]. Our analysis reveals that all of these proteins are highly hydrophilic, with values of over about 1 and, with the exceptions of COR85, COR47, and ERD10, all have glycine contents over 0.08 (Table 4). It is likely that other cold-responsive proteins with similar properties are also involved in plant freezing tolerance. Therefore, we propose that the existing COR/LEA genes be classified based on these properties. Group 1 represents COR/LEA proteins generally meeting the definition of hydrophilins [24] with hydrophilicity over 1 and glycine contents over 0.08. Group 2 represents highly hydrophilic proteins that have glycine contents less than about 0.07 (see Table 2). There are eight proteins from the first group and three from the second that have evidence to support a functional role in plant freezing tolerance. Group 3 hydrophilic proteins, with hydrophilicity less than 1 and glycine contents over about 0.07 could also be retained, as it contains many named COR/LEA proteins. However, no members of this group have been shown to be important for plant freezing tolerance. Of the named unknown proteins reported to be unusually hydrophilic COR8.5, COR28, and COR8.6 fit into groups 1, 2, and 3 respectively [6]. Two additional named unknown proteins, COR12 (± signal peptide) and COR18 and eight of the 11 transiently expressed genes encoding COR/LEA-like hydrophilic proteins [6] do not fit into these groups. The LEA proteins designated by AGI locus codes At1g01470 and At1g16850 and reported as being cold-responsive [12,53] are also not members of these groups.

**Table 4:**
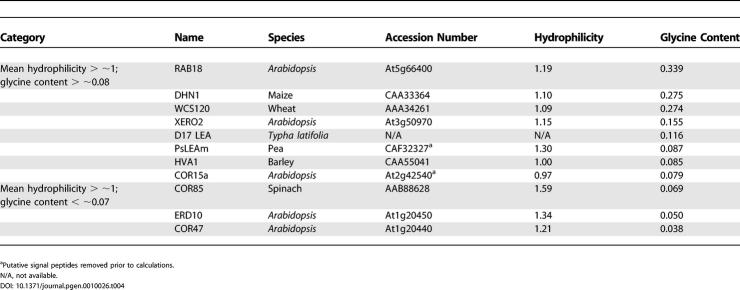
Mean Hydrophilicity and Glycine Content of COR/LEA Proteins Shown to Have Effects that May Be Important for Plant Freezing Tolerance

^a^Putative signal peptides removed prior to calculations.

N/A, not available.

We have separated the known COR proteins (see Table 2) and cold induced unknown proteins (see Table S5) into these groups. A cutoff of 0.3 was used as a lower limit for the hydrophilicity of the unknown proteins, as this is close to the mean of all genes (0.32) and to the minimum for a named COR gene (0.29). Most of the established COR genes are highly up-regulated at multiple time points and have high frequencies of DRE and ABRE elements in their promoter regions (see Table 2). Only *COR28*, *D113*, and *M17* are not up-regulated at multiple time points. We identify 11, 30, and 46 cold up-regulated unknown proteins that are members of group 1, 2, and 3 respectively (see Table S5). Obviously, not all of these genes are COR genes; some genes, such as those designated by AGI locus codes At1g13650 and At1g69160 are down-regulated at other times, while others, such as At1g73350 and At4g17410, are only weakly up-regulated (see Table S5). However, there are many others that provide interesting new candidate COR genes that are up-regulated at multiple time points. Some, but not all, contain DRE elements in their 1,000 bp upstream regions (see Table S5). In support of their involvement in protection against freezing stress, hydrophilins are the most significantly over-abundant group of proteins encoded by cold up-regulated genes (see Table S4).

### The Continued Importance of Short-Term Cold-Responsive Transcription Factors in Regulating Long-Term Changes in Gene Expression

In all our datasets of cold induced genes, there is a significant overabundance of the DRE promoter element that is bound by the CBF/DREB transcription factors [26,27]. The further characterization of the consensus motif for CBF3/DREB1A as A/GCCGACNT [12] is supported by our study, as this motif is significantly overrepresented in the 500-bp promoter regions of all our cold up-regulated gene lists (see Table S4). The A/GCCGACNT motif in the 500–1,000-bp region and the A/GCCGAC motif in the 500-bp region are also overrepresented, but to a lesser extent. The occurrence of the A/GCCGAC motif in the 500–1,000-bp region is not higher than expected. Our data extend the number of cold-responsive genes that are members of the CBF regulon from 38 [12] to a possible 478 genes, representing about 23% of those up-regulated. Included among these genes are 41 known and putative transcription factors, which are mainly from the AP2/EREBP, CO-like, and C2H2 families (S7, M7, and L7 [DRE] in Dataset S1). However, not all of these genes are likely to be under the control of CBF transcription factors, as the frequency of these motifs among all genes is 0.16 and among down-regulated genes 0.145, indicating that the proportion of up-regulated genes in the CBF regulon may be closer to 0.1. Previous work has shown that cold-responsive genes in the CBF regulon respond within 12 h and continue to be up-regulated after 24 h [12], and that many of the genes are stably up-regulated in the long term [6]. We find that the frequency of the DRE element increases for genes up-regulated at multiple time points and is similar among lists for short to medium and medium to long term up-regulated genes.

Overexpression of the CBF transcription factors revealed overlapping and redundant functionality [11]. However, analysis of a mutant in which the *CBF2* gene was disrupted indicated that this gene negatively regulates *CBF1* and *CBF3* and may ensure their expression is transient and tightly controlled [14]. *CBF2* is up-regulated approximately 4-fold in the long term. Although *CBF1* and *CBF3* are not on our long term consensus list, it is still likely they are up-regulated. They are both about 2-fold up-regulated in our ATH1 dataset, and in the other large long-term study [9], they are up-regulated approximately 2- and 8-fold in plants grown on plates and soil, respectively, but are excluded from the final lists due to the MAS5 filtering criteria. These expression levels are much lower than their initial induction, but are still significantly higher than before cold treatment. In addition, 12 other short-term–responsive transcription factors continue to be up-regulated at all times during long-term cold acclimation. These include *RAP2.1, RAV1, ZAT10,* and *COL1,* all of which contain the A/GCCGACNT motif in their 1,000-bp promoter regions. This indicates that some of the regulators that are responsible for the initial large changes of expression may continue to regulate the smaller but stable changes of gene expression that are required to maintain the cold acclimated state.

### Global Analysis Reveals That Many Transcription Factor Families Show Coordinate Regulation

The transcriptional regulation of cold-responsive gene expression is well studied and much is now known. The AP2/EREBP family, with 17 members up-regulated in the short term, has received most attention. The key role of the CBF/DREB transcription factors is well established [6,10,13]. Other members, such as RAP2.1, RAP2.6, and RAV1 have also attracted interest [6]. The significant role of this family in the short-term is supported by our statistical analysis (see Table S6). Our analysis reveals other families that are significantly overrepresented among up-regulated transcription factors that may also be important for cold acclimation. The CO-like family is the most significantly overrepresented family at all times. CO controls the photoperiodic regulation of flowering. CONSTANS-LIKE 1 and 2 (COL1 and 2) are also circadian controlled and *COL1* overexpression influences circadian rhythms and may be light sensitive [54]. The overlap between stress- and circadian-regulated gene expression has been highlighted in previous studies [7]. The overrepresentation of CO-like transcription factors may indicate an important role for these transcription factors in the integration of cold-responsive gene expression with light and circadian regulation.

As has been previously reported, there are also transcription factors down-regulated in the short term [6]. With large numbers of transcripts down-regulated in the medium to long term, it seems likely that some of the 25 short-term and 133 medium-term down-regulated transcription factors also have key roles in the response of plants to low temperature. The significant overrepresentation of transcription factor families among down-regulated transcription factors has not been previously reported and indicates there may be functional overlap in their response to low temperature. The Aux/IAA family is the most affected in the long term and is also significantly overrepresented in the medium term. The bHLH family is significantly overrepresented in the long term. The affected members of these families could provide new insights into processes important in the long-term response to low temperature.

### Changes in Hormone Processes May Be Central to the Regulation of Growth during Cold Acclimation

The hormone functional group is the only group overrepresented among down-regulated genes at all time points, indicating a functional relevance of altered hormonal regulation during the process of plant cold acclimation. Auxin-induced genes are most abundant, although the metabolism, signaling, and response of other hormones are also affected. It has previously been shown that there is cross-talk between cold- and ABA-regulated gene expression, but that the accumulation of ABA is transient, peaking at 24 h in the cold [3]. Based on the timing of expression changes, it was concluded that there may also be cross-talk with ethylene responses, but that little ethylene was likely to be produced [6]. These responses both involve short-term up-regulation of gene expression, while our analysis reveals the importance of down-regulation. This is most likely related to the decreased growth observed in response to low temperature, but whether the down-regulated genes are causal in this relationship is uncertain. As many metabolites increase in response to low temperature [13,34,35], resource limitation of growth is unlikely. This leads to the question of which mechanisms are responsible for regulating growth. Changes in the expression of enzymes of hormone metabolism and coordinate changes among hormone responsive transcription factor families support the role of hormones in this regulation.

A phylogenetic analysis based on bHLH domain amino acid sequences revealed 21 sub-families of bHLH transcription factors [55]. The sub-family 18 is the largest, containing 16 of the 117 genes from our database belonging to these families including *BEE1, BEE2,* and *BEE3,* which are redundant brassinosteroid (BR)-responsive genes that are also repressed by ABA [56]. This family contains two of the nine medium-term (AGI locus codes At3g07340 and At3g23690) and three of the nine long-term (*BEE2* and AGI locus codes At5g39860 and At5g50915) bHLH transcription factors down-regulated in response to cold. Our data indicate that *BEE1, BEE3,* and AGI locus codes At2g18300 and At3g23690 are also long-term down-regulated, although these differences are significant for only one of our two datasets. Additionally, analyses of raw ATH1 data indicate that many of these changes are likely to begin at 24 h (unpublished data). A survey of the AtGenExpress hormone data series reveals that genes designated by AGI locus codes At2g18300 and At5g39860 are strongly down-regulated by ABA and At5g50915 is up-regulated by brassinolide after 3 h [57].

Members of the Aux/IAA family of transcription factors, which are up-regulated by auxin, repress the interaction of ARF family proteins with target genes, leading to either activation or repression of transcription [58]. Among our long-term cold-responsive genes, the Aux/IAA family is coordinately down-regulated and the ARF family is up-regulated. The up-regulation of Aux/IAA and down-regulation of ARF genes have been reported in response to BR treatment [59]. Moreover, it has been suggested that the Aux/IAA transcription factors may be important cross-talk points in BR, auxin, light, and other signaling pathways [60].

The cross-talk between auxin and BR may be more extensive; a recent study reported a 25% overlap between the auxin- and BR-induced transcriptomes [59]. There are 332 genes represented in our database that are reported to be up-regulated in response to BR [59]; of these, 46 and 38 are significantly down-regulated in the medium and long term, while the corresponding up-regulated lists contain only 19 and 11 of these genes, respectively. Of a similar number of auxin up-regulated genes there are 10 and 28 long-term up- and down-regulated in response to cold; however, 11 of the down-regulated genes also respond to BR [59]. The number of BR up-regulated genes down-regulated by cold is about 2.8-fold the number expected by chance, and includes ten genes annotated as auxin-responsive proteins. Some of these are members of the Aux/IAA transcription factor family. Two genes are annotated as being involved in gibberellin processes and three are GDSL-lipases. An alternative study reported less overlap between IAA- and BR-induced genes, but of the 31 genes up-regulated by BR and auxin [60], six are down-regulated in the medium term in response to cold. The AtGenExpress hormone data indicate that many of the genes down-regulated by cold and up-regulated in response to auxin and BR are also negatively regulated by ABA [57]. As previously suggested [59], these shared genes may represent a common growth signature, and their down-regulation could also be correlated with reduced growth in response to low temperatures.

The analysis of hormone biosynthetic pathways indicates that any reduction in auxin levels does not appear to be controlled at the transcriptional level, as the only change for genes involved in auxin biosynthesis is the medium-term up-regulation of two enzymes (see Table S3B). There are 16 annotated genes encoding enzymes of the brassinolide biosynthesis pathway, of which five are significantly down-regulated in the long term (see Table S3B). Of these, *DWF1* is also down-regulated in the medium term, and *STE1* in both the short and medium term. Additionally, the cytochrome P450 gene *CYP90A1* is down-regulated in both the short and medium term, and the recently reported *CYP90C1* [61] is medium-term down-regulated. These changes are obviously responsible for the significant overrepresentation of BR biosynthesis among short- and long-term down-regulated genes, and they contribute to the observed overrepresentation of lipid metabolism among long-term down-regulated genes (see Tables 1 and S3A). Of the five annotated enzymes of ABA synthesis, the one designated AGI locus code At3g14440 is highly induced in both the short and medium term.

These findings indicate that ABA may have a significant role in controlling longer-term responses to low temperature. The down-regulation of many BR-responsive genes, the known effect of BR on cell division and expansion [62], and the significant down-regulation of genes involved in BR synthesis indicate that reduced BR signaling may have a central role in reducing growth at low temperatures. The Aux/IAA and bHLH families of transcription factors contain a number of candidates for the regulation of these responses.

In conclusion, the data discussed in this paper provide the most comprehensive overview of cold-responsive gene expression available. Our analyses provide a statistical basis for many previously reported changes and, moreover, identify which processes predominate during cold acclimation. We believe that this approach offers the fullest characterization possible using expression profiling of a single genotype exposed to low temperature. The next significant advance will be to more fully integrate changes of gene expression with changes detected using metabolic profiling. However, approaches using *Arabidopsis* lines with quantitative differences in freezing tolerance due to transgenic or natural genetic variation will be crucial to identify the processes that are central to plant freezing tolerance.

## Materials and Methods

### Plant material.

The *A. thaliana* accession Columbia (Col-0-G1) [63] was grown in soil in a greenhouse with supplementary light providing a 16 h photoperiod at a minimum of 200 μmol m^−2^ s^−1^, and at a day/night temperature of 20 °C/18 °C until bolting (43–46 days after germination). For cold acclimation, plants were transferred to a 4 °C growth chamber with a 16-h photoperiod at 90 μmol m^−2^ s^−1^ for an additional 14 d [64]. Whole rosettes pooled from ten plants were harvested after approximately 8 h of light. The experiment was repeated to give three independent biological replicates.

### Expression analysis.

Extraction, labeling, hybridization, and scanning for the ATH1 arrays was as described previously [17]. Each biological replica was hybridized to a single ATH1 array at the RZPD Deutsches Resourcenzentrum für Genomforschung, Berlin, Germany (http://www.rzpd.de). Aliquots from the same RNA extracts were processed and hybridized to the MWG arrays as described (http://www.THE-MWG.com). All samples were labeled with Cy3 and single-channel hybridized to two MWG arrays to give two technical replicates. All data are available in the TAIR database (http://www.arabidopsis.org) under the submission number ME00369.

### ATH1 data preprocessing.

Data were analyzed using the bioconductor software project [65]. The starting point for all analyses was the “.CEL” files from the MAS5 software. Data quality was assessed using functions in the affy [66] and affyPLM packages. The GCRMA algorithm (ver. 1.1.0) was used to obtain expression estimates [67]. The array element mappings of Affymetrix probe set IDs to AGI locus codes by TAIR (01/06/04) were used. Only probe sets from the ATH1 array that matched either a single locus code or two with clearly matching annotations were included in further analyses (21,581 probe sets). Only probe sets from the AtGenome that matched a single locus code were used (7,097 probe sets) when compiling the database.

### MWG data preprocessing.

Data were obtained from MWG hybridization service (http://www.THE-MWG.com). Briefly, MWG arrays were scanned at multiple photomultiplier amplification settings (PMT gain) and Imagene intensities were combined and log scale normalized using the MAVI Pro software. Spots flagged as low quality and those not matching to an AGI locus code were excluded from further analyses. Data quality of raw and normalized data was assessed using standard functions of the R software (http://www.r-project.org).

### Statistical testing for differential expression.

The two datasets were analyzed separately using the Limma bioconductor package. A linear model was fitted for each gene with the “NA” expression acting as an intercept and the contrast “ACC-NA” as the coefficient of interest. The “duplicateCorrelation” function was used for the MWG technical replication. An empirical Bayes approach was used to moderate t-statistics [32], and an FDR correction for multiple testing was used [20].

### Database construction.

A database of previously reported cold-responsive genes in *Arabidopsis* was compiled from the literature and publicly available datasets (http://affymetrix.arabidopsis.info/; http://www.arabidopsis.org/info/expression/ATGenExpress.jsp). Transcripts reported in the literature to be up- or down-regulated in response to low temperature were classified as responding in the short (12 h or less), medium (24–48 h), or long term (over 48 h). Studies using expression profiling were analyzed separately (Table S8). Unless more complete data were available, cutoffs suggested by the authors were used. Updated mappings of AGI locus codes were used where possible and any duplicate values were averaged. ATH1 data were analyzed with the comparison mode of the Affymetrix MAS5 software. All possible pairwise comparisons between cold-treated samples and the appropriate controls were made. Probe sets meeting the Affymetrix suggested criteria (Increase/Decrease, appropriate “present” call, 2-fold change) in all comparisons were considered cold responsive.

### Data analysis.

All protein and nucleotide sequences used were obtained from TAIR. The Web-based TAIR Patmatch (http://www.arabidopsis.org/cgi-bin/patmatch/nph-patmatch.pl) was used to search for cis-elements in promoter regions. Mean hydrophilicity using a sliding window of 12 and glycine content were calculated as described [24] for all *Arabidopsis* genes (TAIR file, ATH1_pep_cm_20040228) using the “seqinr” package and a custom script in the R software. Statistical comparisons of functional groups for cold-responsive genes were made against those for all genes in the database using a Fisher exact test in the R software. Microsoft Excel, Access, and the R software were used to perform general analyses. Unless an alternative value is stated the term significant is used to denote an FDR-corrected *p* < 0.05 for differential expression and an uncorrected *p* < 0.05 in all other cases.

## Supporting Information

Dataset S1 Mapman Visualization of Cold-Responsive Gene Expression See readme.txt_ in ZIP file for Mapman software details. The Short (S), Medium (M), Long (L), SM, ML, and SML files contain the mean log2 expression changes for the consensus genes regulated at these times. The files with the prefix DRE indicate which of these genes contain either the A/GCCGACNT motif in their 1,000-bp promoter or the A/GCCGAC motif in their 500-bp promoter. The All_AGI file includes all 22,043 genes in our database. The suffixed numbers in our manuscript refer to different pathways that can be used for visualization; 1, Cellular response; 2, Secondary Metabolism; 3, Regulation; 4, Transport; 5, Large enzyme families; 6, Photosynthesis; 7, Transcription; 8, Sucrose-Starch; 9, Glycolysis; 10, TCA; and 11, Metabolism.
(3.6 MB ZIP)

Table S1 Comparisons between All Independent Datasets Incorporated into Table S2
Comparisons shown are of the agreement (A), the proportion of common genes (B), and the number of common genes (C) between all independent datasets incorporated into Table S2. Data from grouped datasets were averaged. The agreement is the proportion of common cold responsive genes that change in the same direction in both studies. Comparisons within each time class with greater than 0.7, 0.8, and 0.9 agreement are highlighted yellow, orange, and red, respectively. Studies with fewer than ten common genes are in italics and not highlighted.
(59 KB XLS)

Table S2 Database of Cold-Responsive Genes in *Arabidopsis*
“AGI” contains a single entry for each AGI locus code, while “AGI-2” shows additional matches for ATH1 probe sets. “Probe set ID” shows the ATH1 probe set IDs that match each locus code, and “Probe set ID-2” shows additional matches where present. “AtGenome” and “MWG” indicate genes represented on these arrays; Y, AGI match; S, AGI-2 match; U, no match to ATH1 array; a blank indicates that the gene is not represented. The “Lit” columns contain numbers that correspond to supporting papers (Table S9), while the other columns contain the log2 change of expression for genes considered to be cold responsive in each study. The log2 nonacclimated signal, log2 change, unadjusted, and FDR adjusted *p*-values are provided for both our datasets. Datasets are split into the time classes of short, medium, and long term, and highlighted in yellow, blue, and green respectively. The cold responsiveness of each gene was calculated by scoring 1 for up-regulation and −1 for down-regulation in biologically or technically independent studies in each time class. The average log2 fold change within each time class for genes scoring 2 or above or −2 or below is given in the “_up” and “_down” columns, respectively. The mean hydrophilicity, glycine content, and frequencies of the DRE and ABRE promoter elements are also included. Details of the expression profiling studies used can be found in Table S8.
(13 MB XLS)

Table S3 Statistical Analysis of TAIR AraCyc Pathways and the Table S2 Data for Each Gene Statistical analysis is presented for (A) TAIR AraCyc pathways and (B) the corresponding entries from Table S2 for each gene. All genes from the AraCyc pathways included in aracyc_dump_20040520 (ftp://ftp.arabidopsis.org/home/tair/Pathways/) were used to extract values for cold responsive genes from Table S2. Enzymes without known AGI locus code matches or duplicate codes within a single pathway were excluded. The numbers of genes within each pathway up- or down-regulated in the short, medium, and long term were counted. The proportion of genes within a pathway that are cold responsive was compared to the proportion of all genes that are cold responsive. The fold change of this comparison (+/−) and a *p*-value (p-val) from a Fisher exact test are given. Fold changes of 0 and *p*-values of 1 are not shown, and *p*-values less than 0.05, 0.01, and 0.001 are highlighted in yellow, orange, and red, respectively.
(977 KB XLS)

Table S4 Statistical Analysis of Hydrophilicity and Glycine Content of Proteins Encoded by Consensus Cold-Responsive Genes Hydrophilicity and glycine content were calculated as described [24] and cross-referenced against Table S2 for the all *Arabidopsis* proteins (ATH1_pep_cm_20040228; ftp://tairpub:tairpub@ftp.arabidopsis.org/home/tair/Sequences/blast_datasets/). Where multiple gene models were predicted only the first was used. The proportion of each group cold responsive was compared to the proportion of all genes within our database that are cold responsive. The fold change of this comparison (+/−) and a *p*-value (p-val) from a Fisher exact test are given; *p*-values less than 0.05, 0.01, and 0.001 are highlighted in yellow, orange, and red, respectively. Hydrophilins are defined as having hydrophilicity values over 1 and a glycine content over 0.08 [24].
(21 KB XLS)

Table S5 Mean Hydrophilicity and Glycine Content of Unknown Proteins that May Be COR/LEA Proteins Genes annotated as expressed or hypothetical proteins up-regulated in the short, medium, or long term were selected. The mean expression changes during short, medium, and long term cold treatment and the frequencies of the DRE and ABRE cis-elements are also shown. Mean hydrophilicity was calculated using a sliding window of 12 residues as described [24]. Proteins are grouped by their hydrophilicity and glycine content and sorted by AGI code. AGI locus code At2g36220 has been previously reported as COR/LEA-like [6]. Although not unknown, At3g02480 is also included as it has not been previously reported.
(30 KB XLS)

Table S6 Statistical Analysis of the Cold Regulation of Selected Transcription Factor Families The proportion of each family that is cold responsive was compared to the proportion of all transcription factors within our database that are cold responsive. The fold change of this comparison (+/−) and a *p*-value (p-val) from a Fisher exact test are given; *p*-values less than 0.05, 0.01, and 0.001 are highlighted in yellow, orange, and red, respectively. The numbers of genes in each family and the total numbers of transcription factors regulated are also shown.
(21 KB XLS)

Table S7 Table S2 Extract for Housekeeping Genes Used in this Study This table includes all actins, elongation factors, DNA-dependent RNA polymerases and poly-ubiquitins, tubulins not reported as cold responsive [29], and annotated cytosolic cyclophilins. Only one AGI locus code entry was retained for ATH1 probe sets that cross-hybridize to close family members. See Table S2 for more details.
(45 KB XLS)

Table S8 Details of the Studies Used to Compile Table S2
Datasets are listed in the first row with experimental, analysis, and reference details below. Biologically or technically independent datasets within each time class are grouped. “Growth” indicates the growing medium and “Age” the age or growth stage of plants prior to cold treatment. “Time,” “Photoperiod,” “Light intensity,” and “Temperature” refer to the cold treatment conditions. Details regarding “Array” and “Analysis” can be found in Materials and Methods. In “Replication,” T, technical; B, biological; and −, “hybridized with...”. “Data” provides a link to the dataset used, and “Reference” the relevant publication.
(31 KB XLS)

Table S9 Publications Detailing Experimental Evidence for Cold-Responsive Gene Expression The numbers in the “Down” and “Up” columns correspond to those included in the “Lit” columns of Table S2, and a supporting publication is given in the “Reference” column. Genes were identified by AGI locus code, accession number, gene name, or a high-scoring BLAST match. In a few cases where the probes used would cross hybridize, very similar family members were included.
(40 KB XLS)

### Accession Numbers

The Institute for Genomic Research (http://www.tigr.org) AGI locus codes for the genes and proteins discussed in this paper are: *ABA-responsive protein similar to KIN1 from Brassica napus* (At3g02480), *alcohol dehydrogenase* (At1g77120), *APY2* (At5g18280), *AtGolS3* (At1g09350), *BEE1* (At1g18400), *BEE2* (At4g36540), *BEE3* (At1g73830), *CBF1* (At4g25490), *CBF2* (At4g25470), *CBF3* (At4g25480), *COL1* (At5g15850), *COL2* (At3g02380), *COR12* (At4g33550), *COR18* (At2g43060), *CYP90A1* (At5g05690), *CYP90C1* (At4g36380), *DREB2B* (At3g11020), *DWF1* (At3g19820), *lactate dehydrogenase* (At4g17260), *P5CS2* (At3g55610), *pyruvate decarboxylase* (At4g33070), *RAP2.1* (At1g46768), *RAP2.6* (At1g43160), *RAV1* (At1g13260), *RCI2A* (At3g05880), *RCI2B* (At3g05890), *STE1* (At3g02580), *ZAT10* (At1g27730), and *ZAT12* (At5g59820).

## Abbreviations

ABA: abscisic acid
ABRE: abscisic acid response element
AGI: *Arabidopsis* Genome Initiative
AP2/EREBP: APETALA2/ethylene response element binding protein
ARF: auxin response factor
AtGenome: *Arabidopsis* Genome Array
ATH1: Affymetrix ATH1 genome array
bHLH: basic helix-loop-helix
BR: brassino-steroid
CBF: C-repeat binding factor
CO: CONSTANS
COL: CONSTANS-LIKE
COR: cold-regulated
DREB: dehydration-responsive element binding
FDR: false discovery rate
HSF: heat shock transcription factor
LEA: late embryogenesis abundant
MAS5: Microarray Suite 5
MWG: MWG Biotech 25k *Arabidopsis* 50-mer oligonucleotide array
TAIR: The *Arabidopsis* Information Resource
TCA: tricarboxylic acid
